# The long-term rate of change in lung function in urban professional firefighters: a systematic review

**DOI:** 10.1186/s12890-018-0711-8

**Published:** 2018-09-06

**Authors:** Flynn Slattery, Kylie Johnston, Catherine Paquet, Hunter Bennett, Alan Crockett

**Affiliations:** 1 Alliance for Research in Exercise, Nutrition and Activity, Sansom Institute for Health Research, School of Health Sciences, Universitiy of South Australia, Adelaide, Australia; 2 School of Health Sciences, Sansom Institute for Health Research, University of South Australia, Adelaide, Australia; 3 Centre for Population Health Research, Sansom Institute for Health Research, School of Health Sciences, University of South Australia, Adelaide, Australia

**Keywords:** Firefighters, Firefighting, Spirometry, Lung function, Exposure, Longitudinal, Systematic review

## Abstract

**Background:**

Despite the known occupational hazards, it is not yet clear whether long-term career firefighting leads to a greater rate of decline in lung function than would normally be expected, and how this rate of change is affected by firefighting exposures and other risk/protective factors.

**Methods:**

A systematic search of online electronic databases was conducted to identify longitudinal studies reporting on the rate of change in the forced expiratory volume in one second (FEV_1_) of forced vital capacity (FVC). Included studies were critically appraised to determine their risk of bias using the Research Triangle Institute Item Bank (RTI-IB) on Risk of Bias and Precision of Observational Studies.

**Results:**

Twenty-two studies were identified for inclusion, from four different countries, published between 1974 and 2016. Examined separately, studies were categorised by the type of firefighting exposure. Firefighters experienced variable rates of decline in lung function, which were particularly influenced by cigarette smoking. The influence of routine firefighting exposures is unclear and limited by the methods of measurement, while firefighters exposed to ‘non-routine’ severe exposures unanimously experienced accelerated declines.

**Conclusions:**

The data provided by longitudinal studies provide an unclear picture of how the rate of change in lung function of firefighters relates to routine exposures and how it compares to the rate of change expected in a working-age population. Non-smoking firefighters who routinely wear respiratory protection are more likely than otherwise to have a normal rate of decline in lung function. Exposure to catastrophic events significantly increases the rate of decline in firefighter lung function but there is limited evidence detailing the effect of routine firefighting. Future studies will benefit from more robust methods of measuring exposure.

**Trial registration:**

International Prospective Register of Systematic Reviews (PROSPERO), registration number (CRD42017058499).

## Background

The risks to firefighters’ respiratory health are well known. Reductions in lung function, increases in airway hyper-responsiveness, and the onset of other symptoms of respiratory illness have been reported in firefighters following exposures during firefighting duties [1–6].

Other reports indicate that firefighters have better lung function than the general population in both FEV_1_ and FVC: likely due to a strong healthy worker effect [7–10]. This makes the routine comparison of these values to a reference standard following a single pulmonary function test more challenging, and may serve to misclassify some firefighters’ lung function. For example, a firefighter with an FEV_1_ of 5.0 l (and 130% of predicted) could lose more than 1 litre before being below 100% of predicted normal, and more than two litres before being below the lower limit of normal (LLN) [11]. Serial measurements and subsequent analyses of the rate of change in lung function may represent the most useful way of monitoring firefighter respiratory health.

The long-term rate of change in FEV_1_ in healthy, non-smoking adults of working age was initially reported by Fletcher and Peto as − 36 mL/yr [12]. Further studies have reported rates of change ranging from around − 20 to − 38 mL per year [13–21], and as much as 56 mL per year [22]. Despite the known occupational hazards, it is not yet clear whether long-term routine firefighting leads to a greater rate of decline in lung function than would normally be expected. This review aims to answer the following questions: 1) What is the rate of change of lung function in professional urban firefighters? 2) How is this rate of change influenced by level of exposure to routine firefighting and non-routine firefighting (i.e. catastrophic events) and protective or deleterious factors? 3) How is the rate of change in lung function measured/calculated and reported in studies of professional firefighters?

## Methods

This systematic review was conducted in accordance with the Preferred Reporting Items for Systematic Reviews and Meta-Analyses (PRISMA) Statement guidelines [23], and the protocol was registered on the International Prospective Register of Systematic Reviews (PROSPERO) (registration number CRD42017058499).

### Selection of studies

Studies selected for review had to satisfy three conditions: 1) FEV_1_ and/or FVC had to be measured in the same individuals on more than one occasion (if not using regression techniques), with a minimum observation period of 12 months; 2) The rate of change in either FEV_1_ or FVC had to be available directly or calculable from the presented data; and 3) Participants had to be adult (≥ 18 years of age) full-time professional urban firefighters; excluding part-time, volunteer and country/wildland firefighters. There was no restriction placed on publication date or language.

### Search strategy

Relevant publications were initially sought with a systematic search conducted on March 8 2017, using the online electronic databases CINAHL, Embase, Medline, Medline (Epub ahead of print), Scopus and Web of Science. Under the advice of an academic librarian, the following keyword string was used to find candidate papers: ((“fire fighter*” or firefighter* or firem#n or “fire m#n”) or (fire [within three words] personnel)) AND ((“lung* function” or “pulmonary function” or respiratory) or (FEV* or “forced expiratory volume*” or FVC* or “vital capacit*” or spirometr*)). When available, the following subject headings were also combined with the keyword search (Firefighters/) AND (Lung/ or spirometry/ or vital capacity or forced vital capacity or forced expiratory volume or respiratory airflow). Two authors independently conducted all searches, collated all returned titles and abstracts and removed duplicate items.

### Title and abstract screening

All titles and abstracts were independently screened to assess each item’s suitability for full-text review. When the title or abstract provided insufficient information to make a decision, the full-text paper was retrieved. The authors then independently reviewed all selected full-text papers and selected eligible papers for inclusion. Reference lists and citations (Google Scholar search March 29 2017) of eligible papers were then screened and the full-texts of relevant papers were examined: eligible papers were then included for review. Discrepancies were resolved at each stage of the selection process by discussion between the two authors, with a third author available for adjudication in case of disagreement.

### Data extraction

Data from each included paper were independently entered into a database by two authors. Extracted information included, but was not limited to, the characteristics of the cohort(s) studied, study methodology and results. When the data were only reported graphically, they were extracted using an online tool [24]. When the rate of change in FEV_1_ and/or FVC was not reported and unavailable from the authors, it was calculated (and rounded to the nearest whole millilitre) as the difference between baseline and follow-up value divided by the time interval (or when more than two data points were available: calculated by using simple linear regression). When available, the respective rates of change were reported stratified by smoking status as well as for the entire cohort. When stratified data were not available, and the average rate of change for the entire cohort was reported alone, as well as the cohort’s smoking rate.

### Quality assessment

Included studies were critically appraised to determine their risk of bias using the Research Triangle Institute Item Bank (RTI-IB) on Risk of Bias and Precision of Observational Studies [25], which provides a means to assess the quality of studies related to exposure outcomes. The RTI-IB is one of the only quality appraisal scoring tools available for observational studies, providing a comprehensive list of 29 questions covering a range of categories of bias [26]. The authors recommend the tool be modified based on its appropriateness to the literature. For this reason, questions 8, 12, 26 and 27 of the tool were omitted, due to their inapplicability to the topic, while a “cannot determine” response was added to question 13. The critical appraisal was carried out independently by two authors, with discrepancies being resolved by discussion. Each study was given a score based on the number of applicable RTI-IB items met and subsequently graded, based on previous publications [27–29] as low (0–.40), moderate (.41–.70), or high (.71–1) methodological quality/risk of bias.

### Data analysis

A descriptive analysis was conducted due to the large heterogeneity of the included studies in terms of their population characteristics, type of assessment of exposure, and reporting of outcome measures.

## Results

**Fig. 1:**
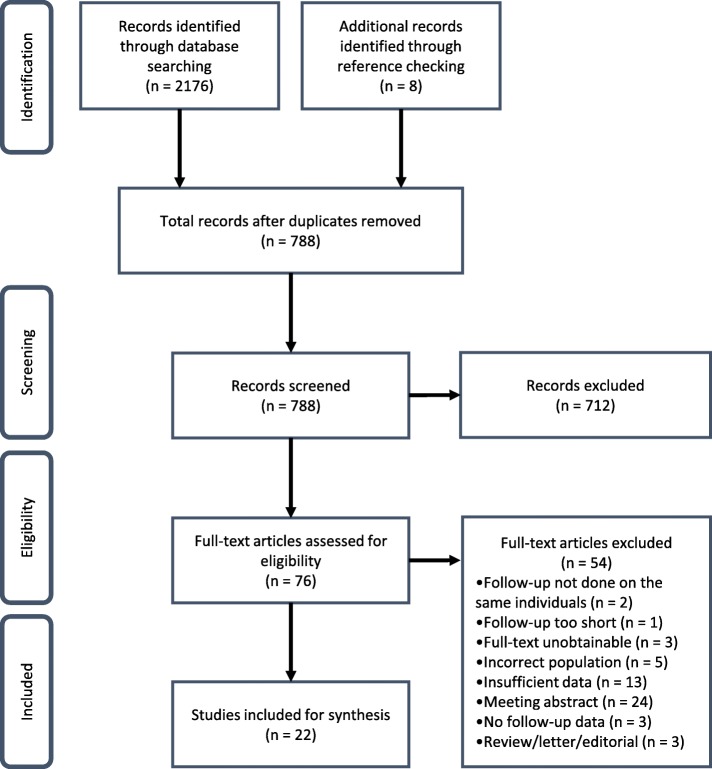
PRISMA flow diagram of included and excluded studies

The searches yielded a total of 788 unique articles, including eight that were identified through reference checking (Fig. 1). Following the screening and review process, a total of 22 papers met the eligibility criteria and were included for review.

### Characteristics of included studies

Descriptive information about the included studies is summarised in Table 1 and includes study location and dates, the baseline characteristics of the study population and the methods of conducting spirometry and measuring exposure.

**Table 1:** Descriptive information. Studies are ordered by population type and year of publication

Author & Year [Ref]	Location and period	Population (n=)	Baseline age (years)	Race (%)	Sex (%)	Standardisation of spirometry	Measurement of exposure (main index)
Populations exposed to routine firefighting							
Peters et al. 1974 [37]	Boston, USA 1970 to 1972	Firefighters (1430)	43.13	NR	M	Average of best 3 of 5 trials	Interview using structured questionnaire (fires fought in previous 12 months)
Musk et al. 1977 [35]	Boston, USA 1970 to 1974	Firefighters (1146)	41.9	NR	M^a^	Average of best 3 of 5 trials	Interview using structured questionnaire and BFD records (fires fought in previous 12 months, service time)
Musk et al. 1977 [36]	Boston, USA 1970 to 1975	Retired firefighters (109)	54.5	NR	M^a^	Average of best 3 of 5 trials	Interview using structured questionnaire (fires fought in 12 month period, service time)
Musk et al. 1982 [34]	Boston, USA 1970 to 1976	Firefighters (951)	40.9 (9.4)	W	M^a^	Mean of best 3 of 5 satisfactory (within 5% of best trial) trials	Interview using structured questionnaire and BFD records (fires fought in previous 12 months)
Douglas et al. 1985 [31]	London, England 1976 to 1977	Firefighters (890)	25-29^b^	NR	M	≥ 5 FVC manoeuvres, mean of the last 3 values used for analysis	Self-report questionnaire (service time, absence from work after exposure)
Tepper et al. 1991 [33]	Baltimore, USA 1974–77 to 1983–84	Firefighters (628)	38.2 (10)	C (86)	M	ATS 1978	Estimated from fire department records (years spent in exposed jobs before baseline, number of emergency responses before baseline) and self-report questionnaire (previous exposure to ammonia/chlorine)
Kales et al. 1997 [38]	Boston, USA 1992–93 to 1995	HAZMAT firefighters (37)	36.8 (5.9)	NR	M	ATS 1979	NR
Burgess et al. 2004 [40]	Phoenix, USA 1988 to 1999	Firefighters (1204)	34.6 (8.9)	W (75), H (16), B (6), O (3)^c^	M (96) F (4)	No info available; retrospective analysis of existing database.	None. Retrospective analysis of existing database.
Josyula et al. 2007 [41]	Phoenix, USA 1998 to 2005	Firefighters (67)	38.6 (7.8)	W (78), H (10), AA (6), O (6)	M (96) F (4)	ATS 1987	Self-report questionnaire (not used in analysis)
Yucesoy et al. 2008 [42]	Phoenix, USA 1988 to 2003	Firefighters (374)	M: 31.9 (6.4) F: 29.7 (3.9)	M: NHW (76.4), HW (19.5), AA (4.1). F: NHW (100)	M (97.3) F (2.7)	ATS 1987	None
Populations exposed to routine firefighting with non-firefighter controls							
Sparrow et al. 1982 [10]	Boston, USA 1963–68 to 1968–1973	Firefighters (168)^d^ GP controls (1474)^d^	NR	NR	M	Best 1 of 3 ‘acceptable’ tracings (≥4 s with maximal effort)	Self-report questionnaire (service time)
Horsfield et al. 1988 [32]	West Sussex, England NR	Firefighters (96) GP controls (69)	32.5 [Range 18–54]^e^ 39.5 [Range 16–63]	NR	M	NR	None
Hnizdo 2012 [43]	Phoenix, USA 1989 to 2000	Firefighters (965) Paper-pulp mill workers (1286) Construction workers (460)	36.3 (9.3) 36.4 (8.4) 35.4 (8.8)	NR	M	ATS 1994	None. Retrospective analysis of existing database.
Aldrich et al. 2013 [9]	New York, USA 2003–06 to 2011	Firefighters (940) EMS controls (97)	26.1 (3.3) 27.6 (7.0)	B (6), W (94) B (52), W (48)	M	≥ 3 acceptable efforts with standardised criteria	None
Schermer et al. 2013 [8]	Adelaide, Australia 2000–08 to 2003–2011	Firefighters (254) GP controls (678)	43.5 (8.0) 43.4 (9.8)	C (99.6) C (95.5)	M	Firefighters: ATS/ERS 2005 Controls: ATS 1987	Self-report questionnaire (use of respiratory protection)
Choi et al. 2014 [30]	Daegu, Korea 2008 to 2011	Firefighters (322) Non-firefighter controls (107)	43.6 (6.9) 44.1 (10.1)	NR	NR	≥ 3 acceptable efforts with standardised criteria	Interview by physician using structured questionnaire (active/inactive firefighting status)
Populations exposed to non-routine firefighting							
Unger et al. 1980 [39]	Houston, USA 1987 to 1989	Firefighters exposed to major chemical warehouse fire (20)	27.2 (5.36)^f^	B, W	M	Best of 3 trials	Self-report questionnaire at 6-week follow-up
Banauch et al. 2006 [44]	New York, USA 1997 to 2002	9/11-exposed FDNY firefighters & EMS workers (11766)	39.7 (7.7)^g^	W (85.6)	M (95.6), F (3.4)	ATS 1994	Self-reported arrival time at WTC site
Aldrich et al. 2010 [45]	New York, USA NR to 2008	9/11-exposed firefighters (10870) 9/11-exposed EMS workers (1911)	40.8 [CI, 40.6–40.9)^g^ 37.1 [CI, 36.7–37.5]^g^	W (94), B (2.5) W (49.7), B (22.2)	M (99.8), F (0.2) M (75.6), F (24.4)	ATS/ERS 2005	Self-reported arrival time at WTC site
Banauch et al. 2010 [48]	New York, USA 2001 to 2005	9/11-exposed firefighters (90)	40.7 (7.1)^g^	W (86)	M (98), F (2)	ATS/ERS 2005	Self-reported arrival time at WTC site
Aldrich et al. 2016 [46]	New York, USA 2000 to 2014	9/11-exposed firefighters (10641)	41.4 [Range 21.3–74.6]^g^	W (97.4), AA (2.6)	M (99.8), F (0.2)	ATS/ERS 2005	Self-reported arrival time at WTC site
Aldrich et al. 2016 [47]	New York, USA 2000 to 2014	9/11-exposed firefighters (173)	42.6 (7)^g^	W (95.4), AA (4.6)	M	NR	Self-reported arrival time at WTC site

Values are means (SD), unless stated otherwise. 9/11 = World Trade Center disaster on September 11, 2001, *AA* African-American, *ATS* American Thoracic Society, *B* Black, *BFD* Boston Fire Department, *C* Caucasian, *CI = 95%* Confidence interval, *EMS* Emergency Medical Services, *ERS* European Respiratory Society, *F* Female (s), *FDNY* Fire Department of New York, *GP* General population, *HAZMAT* Hazardous materials, *HW* Hispanic white, *LFB* London Fire Brigade, *M* Male(s), *N* = Total number of participants used in the rate of change analysis, *NHW* Non-Hispanic white, *NR* Not reported, *NWAHS* North-West area health study, *PFT* Pulmonary function test, *ROD* Rate of decline, *USA* The United States of America, *W* White, *WTC* World Trade Center. ^a^Inferred based on timeframe of study, ^b^Median age range (reported in 5-yr intervals), ^c^Estimated based on frequencies within 1400 Phoenix firefighters at the time of the study, ^d^Normative Ageing Study, ^e^Mean of *n* = 101 firefighters measured at follow-up (96 of whom were included for analysis), ^g^Mean of *n* = 24 firefighters measured at baseline (20 of whom followed-p and included in analysis, ^h^Age on 9/11

Within the 22 studies, all published between 1974 and 2016, there were 11 distinct firefighter populations: one from each of Australia [8] and South Korea [30], two from England [31, 32] and the remaining seven from the USA. These seven populations consisted of firefighters from Baltimore [33], Boston in both the 1960/1970s [34–37] and 1990s [38], Houston [39] and Phoenix [40–43], as well as New York firefighters exposed [44–48] or not-exposed to 9/11 [9]. The average age of active firefighters at study commencement ranged from 26.1 to 43.6 years, while recently-retired firefighters of one study [36] had an average age of 54.5 years. Seven studies involved both sexes (the highest proportion of female firefighters was 4%) and the remaining included males only. Eleven studies reported the proportions of different racial groups, with the majority of firefighters in each study (76.4 to 100%) being Caucasian/white and the rest being reported as African-American/black (0 to 6%), Hispanic (0 to 19.5%) or unspecified (0 to 14.4%). Two studies reported race without specifying proportions and nine did not report any racial information. Average follow-up time ranged from one to 12.2 years and each study measured lung function at least once, with the highest average number of measures reported being 10.3. Ten studies performed standardised spirometry based on published criteria, nine performed standardised spirometry (usually best or average of three trials) but not according to published standards and three did not report information on spirometry standardisation. The most common method of estimating firefighting exposure was self-report questionnaire (*n* = 15), and three of these studies combined this with an estimate of exposure based on fire department records (one of which did not use these data during analysis). One study obtained information by interview using a structured questionnaire and the remaining six either did not measure exposure, or did not report any measurement.

### The rate of change in FEV_1_ and FVC

#### Routine firefighting

Sixteen studies reported on firefighter populations involved in routine firefighting (Table 2). Among nine studies reporting FEV_1_ change without stratifying by smoking status (smokers and non-smokers pooled together), six observed declines of between − 24.99 and − 39.6 mL/yr. [33, 35, 40–43] while the remaining three showed declines of − 68.2 to − 110 mL/yr. [30, 31, 37]. Within these nine studies, four included smoking status in their regression modelling: two studies observed significantly greater declines in both ever-smokers relative to never-smokers (additional 4.7 mL/yr. decline, *p* = 0.042) [42] and current smokers relative to non-smokers (Actual difference and *p* value not reported) [31], while the two others reported no significant effect [40, 41]. One study reported different rates of decline when stratified by occupational exposure, but observed no significant differences in smoking habits between the groups [37] while the remaining four studies did not report on the longitudinal effect of smoking on lung function [30, 33, 35, 43]. Five studies reported on the rate of change in FVC without stratifying by smoking status, observing declines of − 16.55 (66.75) [33], -40 [35], -76.7 [37], -103 [30] and − 107 [31] mL/yr. (SD (where available)). Among these studies, one reported significantly greater declines in current smokers relative to non-smokers (*p* value not reported) [31].

**Table 2:** Rate of decline in FEV_1_. Studies are ordered by population type and year of publication. Values are means (SD), medians [IQR] or means [95% CI]

Author [Ref]	Group	Follow-up (yr)	No. measures	Calculation of rate of change (no. adjusted variables)	Whole-group baseline FEV_1_ (L)	Rate of change in FEV_1_		Effect of exposure	Effect of risk/protective factors
						Smoking Status [% smokers]	mL/yr		
Populations exposed to routine firefighting only									
Peters et al. 1974 [37]	Firefighters	1	2	Δvalue/Δtime	3.578	Mix [NR]	-68.2	Significant difference in FEV_1_ changes when stratified by exposure (no. of fires fought in previous 12 months): FEV_1_ change (mL/yr): 1–40 fires; − 49, 41–99 fires; −71, ≥100 fires; −109 (*p* < 0.02).	No apparent differences in age, height, smoking habits, race when compared between groups stratified by exposure.
Musk et al. 1977 [35]	Firefighters	3.4	3	Δvalue/Δtime^a^	3.62	Mix [NR]	-30	No significant relationship between FEV_1_ change and estimated (by fire department records or firefighter) fires fought in previous 12 months. No relationship between FEV_1_ change and fires fought when stratified by age, smoking status or service time. Significantly greater FEV_1_ decline in firefighters who fought fewer fires in 1973 vs. 1970 than those who fought the same number or more (*p* < 0.05). Firefighters who fought no fires experienced greatest decline.	No significant relationship between FEV_1_ decline and age.
Musk et al. 1977 [36]	Retired firefighters	4.4	3	Δvalue/Δtime^a^	3.19	Nev For Cur Total: Mix [31]	-30 -30 -100* (*p* < .05 relative to Nev & For) Total: -50	No significant difference between FEV_1_ change of retired firefighters who were active vs inactive (during 1970) prior to retirement. No significant difference in FEV_1_ decline when stratified by years of service.	Greater FEV_1_ decline in current vs never or ex-smokers (*p* < .05).
Musk et al. 1982 [34]	Firefighters	6	2	Δvalue/Δtime^b^	3.68 (0.64)	Nev For Cur Cur/For cigar/pipe Total: Mix [NR]	-33 (44) -33 (39) -47 (45) -31 (44) Total: -36	Amongst active firefighters; no relationship between FEV_1_ decline and either calculated^c^ or estimated^d^ number of fires fought in previous 12 months Inactive (fought no fires in previous 12 months) firefighters tended to have a higher rate of FEV_1_ decline than active firefighters (significance not tested).	No correlation between change in FEV_1_, or FVC between 1970 and 1976 and the initial level of FEV_1_ in 1970 (*r* = 0.10 for FEV_1_). No relationship between annual change in FEV_1_ and the stated tendency of the subjects to voluntarily wear protective breathing apparatus.
Douglas et al. 1985 [31]	Firefighters	1	2	NR	NS	Mix [NR]	-92	Only cross-sectional effect of exposure reported. Change in FEV_1_ unrelated to service time, or to absence from work after exposure to smoke.	Statistically significant greater FEV_1_ decline among current smokers (Actual difference and *p* value not reported).
Tepper et al. 1991 [33]	Repeating^e^ firefighters (*n* = 492)	6–10	2	Δvalue/Δtime	3.83 (0.68)	Mix [Cur, 50]	-24.99 (61.23)*	Significantly greater adjusted (multiple linear regresion^2, 4, 14, 15, 18, 21^) FEV_1_ decline in active vs inactive repeating^e^ firefighters (− 29.33 vs 0.30 mL/yr) (*p* < .01), but not non-repeaters^e^. Non-significant trend of greater adjusted FEV_1_ decline in those who reported ever vs never being exposed to ammonia (− 38.82 vs − 23.16 mL/yr) (*p* = .06) (amongst all firefighters), but no differences based on past chlorine exposure. No significant relationship between adjusted FEV_1_ decline and years spent in exposed jobs before baseline or number of firefighting responses before baseline.	Greater adjusted FEV_1_ decline in those who reported never vs ever using a mask while extinguishing fires, but only significant in non-repeaters^e^ (− 68.44 vs − 30.90 mL/yr) (*p* = .01). No significant difference in FEV_1_ decline based on mask-use during fire overhaul.
	Non-repeating^e^ firefighters (*n* = 136)					Mix [Cur, 45]	-34.79 (40.00)* (*p* = .03)		
Kales et al. 1997 [38]	HAZMAT firefighters	2.58	2	Δvalue/Δtime	NR	Nev Cur or For Total: Mix [Ev, 38]	-40.69^f^ -68.6^f^ (*p* = .27) Total: -51	NR	No significant difference in FEV_1_ changes between smokers and former/current smokers, or between younger (≤35 years) and older (> 35 years) firefighters.
Burgess et al. 2004 [40]	Firefighters	≥5	≥6	Simple linear regression	4.27 (0.66)	Mix [Ev, 28]	-34 (43)	NR	Rate of FEV_1_ decline increased significantly with baseline FEV_1_ (*p* < .001) and age (relative to reference group ≤30 yrs. of age: 31–40 yrs. (*p* = .006), 41–50 yrs. and > 50 (*p* < .001), but no significant effect of smoking (never vs ever) or sex. TT genotype at IL-10 SNP 1668 was associated with a significantly lower rate of FEV_1_ decline, compared to the AA genotype (*p* = 0.023) (based on a subsample of firefighters with IL-10 SNP information; *n* = 379) (ANOVA^2, 3, 17, 18^).
Josyula et al. 2007 [41]	Firefighters	7	≥4	Simple linear regression	4.16 (0.70)	Mix [For; 18, Ev; 12] (100% CurNS)	-33 (59)	NR	Greater baseline FEV_1_ and asthma associated with greater FEV_1_ decline (*p* = .002 and *p* = .0023, respectively). Weight gain was close to being significantly associated with FEV_1_ decline (*p* = .05). No significant relationship between FEV_1_ change and gender, baseline age, height, baseline body mass index, race or smoking status. Mean FEV_1_ decline significantly lower in those possessing the TT genotype of the IL-10 (819) polymorphism [n = 3, − 125 (27) mL/yr], vs. the CC [*n* = 33, − 20 (61)] or CT genotypes [*n* = 31, − 38 (51)] (*p* = .009). Increased IL-1RA associated with slower FEV_1_ decline (*p* = .025) (Multiple regression^1, 2, 3, 13, 17, 18, 22^).
Yucesoy et al. 2008 [42]	Firefighters	M: 11.8 (2.5) F: 11.6 (2.3)	M: 10.3 (2.1) F: 10.3 (2.2)	Simple linear regression	M: 4.39 (0.63) F: 3.60 (0.43)	M: Mix [19.8] F: Mix [30]	M: -34 (27) F: -38 (20) Total: -34 (30)	NR	Lower rate of FEV_1_ decline in the presence of the TGFβ1–509 TT genotype (*p* = .043) (multiple linear regression^2, 3, 13, 16, 17, 18, 25^). Carrying an A allele at TNFα-308 (*p* = 0.010) and GG genotype at TNFα-238 (*p* = 0.028) was associated with a more rapid rate of FEV_1_ decline. The TNFα-308A/− 238G haplotype was associated with an increased rate of decline compared with the other haplotypes. Ever-smokers had a significantly greater rate of decline (− 4.7 mL/yr) compared with never smokers (*p* = .042). FEV_1_ changes not significantly different by race or gender.
Populations exposed to routine firefighting only with use of non-firefighter controls									
Sparrow et al. 1982 [10]	Firefighters^g^	5	2	Δvalue/Δtime	4.08 (0.073) (Nev)	Nev For Cur	-81.2 (19.2) -68.2 (8.7) -77.9 (8.5)	Non-significant trend of greater FEV_1_ decline (additional 12 ml/yr) in firefighters vs controls (*p* = .054). No significant relationship between years of employment and FEV_1_ decline.	Greater FEV_1_ decline in current vs never smokers (*p* < .001), adjusted for firefighting status. Non-significant difference in FEV_1_ decline in former smokers vs never (*p* = .530). Greater age and baseline FEV_1_ as well as lesser height were associated with greater rates of FEV_1_ decline (*p* < .001).
	GP controls^g^				3.93 (0.029) (Nev)	Nev For Cur	-64.1 (3.9) -62.8 (3.7) -65.2 (3.2)		
Horsfield et al. 1988 [32]	Firefighters	1–4	4–8	Simple linear regression	NR	Nev For Cur	-66.5* (*p* < .05) -53.8* (*p* < .05) -70.5 Total: -65.4* (*p* < .01)	Compared to GP CON, the rate of change in FEV_1_ was significantly less negative in all firefighters (*p* < .01) and never and former smoking firefighters (*p* < .05).	No significant difference in rate of change in FEV_1_ between firefighting smoking groups.
	GP controls					Nev	-100.3* (All *p* values relative to GP controls)		
Hnizdo 2012 [43]	Firefighters	8–11	≥4	Simple linear regression	4.39 (0.64)	Mix [≈5]	-39.6 (29.5)	NR	NR
	Paper-pulp mill workers				4.33 (0.60) 4.11 (0.68)	Nev Mix [60]	-34.3 (33.5) -45.2 (32.2)^h^		
	Construction workers				4.10 (0.7)	Mix [NR]	-48.7 (50.1)		
Aldrich et al. 2013 [9]	Firefighters	5	5	Linear mixed effects modelling (5^2, 8, 13, 21, 22^)	4.4 (0.6)	Nev Nev Ev	-344.8 [CI, -347.3 to -342.3]^i^ -337.6 [CI, -340.4 to -334.8] -336 [CI, -341 to -332]	No significant difference in FEV_1_ change between Firefighters and controls: average difference (Fire - EMS) 0.2 mL/yr. (CI -9.2 to 9.6).	Weight gain and service time independently associated with increased rate of FEV_1_ decline (*p* value not reported).No difference in FEV_1_ decline in ever vs never smokers.
	EMS control				3.9 (0.7)	Nev Nev Ev	-44.6 [CI, -53.2 to -35.5]^i^ -33.8 [CI, -43.7 to -23.8] -29 [CI, -38 to -19]		
Schermer et al. 2013 [8]	Firefighters	2.9 (0.3)	2	Δvalue/Δtime	4.51 (0.66)	CurNS	+ 15.6 (104.0)^j^	The difference in the annual change in FEV_1_ between the younger and older age categories differed between the firefighters and controls (interaction term stage cohort age category: *p* = .040). Firefighters had a lower odds of accelerated FEV_1_ decline compared with population controls (OR = 0.60, CI 0.44–0.83; *p* = .002) (Logistic regression analysis^2, 9, 18^).	Firefighters who reported never or rarely using their respiratory protection during fire knockdown had a higher odds of accelerated FEV_1_ decline compared with those who used it often or frequently (OR = 2.20, CI 1.02–4.74; *p* = .044)
	GP controls	3.5 (1.1)			3.73 (0.70)	CurNS	-27.8 (78.6)^j^		
Choi et al. 2014 [30]	Firefighters	3	2	NR	NR	Mix [Cur, 11.8]*	-110*	No significant difference between active and non-active firefighters (RMANOVA^2, 7, 8, 12, 18^). FEV_1_ decline was significantly greater in firefighters compared to non-firefighters (*p* < .001).	NR
	Non-firefighter controls					Mix [Cur, 42.9]* (*p* < .001)	-67* (*p* < .01)		
Populations exposed to non-routine firefighting									
Unger et al. 1980 [39]	Exposed firefighters	Post exposure: 1.5	2	ROD not reported^f^	Post exposure: 4.003 (0.633)	Mix [NR]	-81.3^f^	NR. No pre-exposure measurements, no comparison to un-exposed controls.	NR
Banauch et al. 2006 [44]	9/11-exposed FDNY firefighters & EMS workers	Pre 9/11: 5 Post 9/11: 1	1–7	Linear random-effects modelling (5^2, 8, 13, 17, 18^)	4.30 [IQR 3.80–4.80]	Mix [29]	Pre-9/11 (Fire & EMS)-31 Post-9/11: Fire; -383 ml [CI, -393 to -374] EMS; -319 ml [CI, -340 to -299]	Significant difference in pre and post-9/11 FEV_1_, within arrival time–based exposure groups (*p* < .001). Trans-9/11 FEV_1_ decline by exposure group: high-intensity exposure; − 388 ml (CI, − 370 to − 406), intermediate-intensity; − 372 ml (CI, − 363 to − 381), low-intensity; − 357 ml (CI, − 339 to − 374 ml) (Significant linear trend in exposure intensity–response, *p* = .048). Significant differences in trans 9/11 loss, according to work assignment (Fire vs EMS) (*p* < .001).	Significant difference in reported ‘frequent’ use of protective mask on arrival day between exposure groups (*p* < .001); no observed protective effect of mask use frequency on adjusted average post 9/11 FEV_1_.
Aldrich et al. 2010 [45]	9/11-exposed firefighters	Post 9/11: 6.1 [IQR, 5.2–6.6]*	5 [IQR, 4–7]	Linear mixed models (4^2, 8, 13, 17^)	Nev: 4.54^k^ For: 4.48^k^ Cur: 4.46^k^	Nev For^l^ Cur^m^	Post-9/11 -26 [CI, -31 to -20]* -38^k^ -43^k^	FEV_1_ decline 6 months post 9/11: FIRE; − 355 ml [CI, − 352 to − 359], EMS; − 272 ml [CI, − 268 to − 276] (*p* = 0.004). FEV_1_ decline 12 months post 9/11: FIRE; − 439 ml [CI, − 408 to − 471], EMS; − 267 ml [CI, − 263 to − 271] (*p* = 0.003). Firefighters, but not EMS workers, with heaviest dust exposure had significantly larger declines of − 371 ml (CI, − 362 to − 380) during the first 6 months and − 585 ml (CI, − 515 to − 656) during the first year than did the other members of the cohort. Last FEV_1_ in the final 2 years for workers who had never smoked, there was a non-significant trend toward an association between the number of months of work at the WTC site after 9/11 and the FEV_1_ value, a decline of 4 ml per month of work (*p* = .07).	NR
	9/11-exposed EMS workers	6.4 [IQR, 5.9–6.7]* (*p* < .001)			Nev: 3.90^k^ For: 3.90^k^ Cur: 3.80^k^	Nev For^k^ Cur^k^	-40 [CI,-42 to -38]* (*p* < .001) -38^k^ -42^k^		
Banauch et al. 2010 [48]	9/11-exposed firefighters	Pre-9/11: 3 Post-9/11: 4	2–10	Mixed linear random effects modelling (9^2, 8, 10, 11, 13, 17, 18, 23, 24^)	Pre-9/11 4.19 (0.68)	Mix [NR]	Post 9/11: No AAT-deficiency: -37 (SE -28 to -45^k^) (adjusted)	Average FEV_1_ reduction of -370 mL due to 9/11 exposure.	Comparing firefighters with different AAT phenotype combinations: Significantly greater rate of post-9/11 FEV_1_ decline in firefighters with mild (− 69 [SE − 41 to -97^k^] mL/yr) and moderate (− 147 [SE − 110 to -184^k^]) AAT-deficiency compared to normal (*p* = .011). Significant trend for decline rate acceleration by AAT phenotype combination (*p* = .003). Significantly greater rate of post-9/11 FEV_1_ decline in Firefighters with Low AAT serum level (− 86 [SE − 66 to -107^k^]) vs normal (*p* = .027).
Aldrich et al. 2016 [46]	9/11-exposed firefighters	Post 9/11: 12.2 [IQR, 11.6–12.6]^q^	9 [IQR, 7–10]	Linear mixed models (5^2, 8, 13, 17, 21^)	Nev: 4.59^k^ For^n^: 4.61^k^ For^o^: 4.52^k^ For^p^: 4.45^k^ Cur: 4.55^k^	Nev For^n^ For^o^ For^p^ Cur	Post-9/11: -26^k^ -31^k^ -33^k^ -37^k^ -48^k^	Among never smokers, firefighters arriving the morning of September 11 had slightly lower average FEV_1_ than lesser exposed firefighters; this difference remained significant during most of follow-up (*p* < .05 for most 6-monthly time intervals)	Body weight at the time of PFT was associated with FEV_1_ (*p* < .05); for each pound of body mass gained, FEV_1_ decline averaged 3.93 mL. FEV_1_ change differed significantly by smoking status (*p* < .001). After first 3 years of follow-up, never smokers had significantly greater FEV_1_ than current smokers and former smokers who quit after September 11. During last time interval, FEV_1_ significantly greater in non-smokers and those who quit before 9/11 than current or former smokers who quit after 9/11. Firefighters quitting smoking before March 10, 2008, had significantly greater FEV_1_ than current smokers during most of the post-September 11 follow-up.
Aldrich et al. 2016 [47]	9/11-exposed firefighters	Post-9/11: 11.5 (0.5)	Pre-9/11: 1 Post-9/11: 2	Δvalue/Δtime	4.28 (0.67)^q^	Mix [Cur 6.4, For 17.9)	Post-9/11: -32 (unadjusted)-36.78 (adjusted in multiple regression model)	Effect of 9/11 exposure on FEV_1_ decline post-9/11 not investigated. Average reduction in FEV_1_ across 9/11–399 (468.3) mL.	15.39 mL/year more rapid adjusted^2, 6, 8, 13, 19, 20^ FEV_1_ decline in those with BHR at follow-up, compared with those without BHR (*p* = .0104). Use of steroids associated with a 13.01 mL/year slower rate of decline, compared with those who never used steroids (*p* = .0197).

*AAT* Alpha-1 antitrypsin, *BHR* Bronchial hyper-reactivity, *CI = 95%* Confidence interval, *Cur* Current smokers, *CurNS* Current non-smokers, *EMS* Emergency medical services, *Ev* Ever smokers, *FEV*_*1*_ Forced expiratory volume in one second, *FIRE* Firefighters, *For* Former smokers, *FVC* Forced vital capacity, *Gp* General population, *IL-10* Interleukin-10, *IL-1RA* Interleukin-1 receptor antagonist, *IQR* Interquartile range, *Knockdown* Fire suppression, *Nev* Never smokers, *OR* Odds ratio, *Overhaul* Clean-up following fire suppression, *RMANOVA* Repeated measures analysis of variance, *SE* Standard error, *SNp* Single nucleotide polymorphism, *TGFβ1* Transforming growth factor β1, *TNFα* Tumor necrosis factor-α. Adjusted variables: ^1^Asthma status, ^2^Age, ^3^Baseline lung function, ^4^Blood type, ^5^Body mass index, ^6^Bronchial hyper-reactivity, ^7^Duration of exposure, ^8^Height, ^9^History of chronic respiratory conditions, ^10^Interaction of smoking with AAT deficiency, ^11^Length of FDNY tenure, ^12^Physical activity, ^13^Race, ^14^Respiratory protection, ^15^Respiratory symptoms, ^16^Root mean square error term, ^17^Sex, ^18^Smoking, ^19^Steroid use, ^20^Trans-9/11 change, ^21^Weight, ^22^Weight change, ^23^Work assignment on September 11, 2001, ^24^WTC exposure intensity, ^25^Years of follow-up. *Significant difference between groups. ^a^Baseline and final follow-up used for calculation of rate of decline, ^b^Longitudinal results of study reported, ^c^Calculated based on fire department records, ^d^Estimated by firefighter, ^e^Firefighters with repeatable/non-repeatable spirometry reported separately. Repeater is defined as an individual whose two highest values for both FEV_1_, and FVC agreed within one-tenth litre or 5% of the highest value at both the baseline and follow-up studies, ^f^Calculated as ΔFEV_1_/ΔTime by review authors, ^g^Study data obtained from the Normative Ageing Study, ^h^Total among all paper-pulp mill workers, ^i^Unadjusted for weight-gain, ^j^Values reported by authors upon request, ^k^Extracted from graph, ^l^Smoked before 9/11, ^m^Smoked after 9/11, ^n^Quit before 9/11/2001, ^o^Quit between 9/11/2001 and 3/10/08, ^p^Quit after 3/10/08, ^q^Last pre-9/11 measure (Fire and EMS)

Six studies reported changes in lung function in firefighters involved in routine firefighting stratified by smoking status. Two studies observed significantly less negative rates of change in FEV_1_ in never smokers than other smoking groups [36, 38] and four studies found no significant differences [8–10, 32]. One study reported an FVC decline in never smokers of − 10 mL/yr. [36], significantly less negative than current smokers, while four others reported rates of change in FVC of − 19 [9], -27 (52) [34], -66, [32] -76.8 (10.7) [10], and + 11.2 (140.3) [8] mL/yr. (SD), with no significant differences compared to other smoking groups.

Six studies compared lung function changes in firefighters involved in routine firefighting to non-firefighter controls [8–10, 30, 32, 43]. One study showed a significantly greater rate of FEV_1_ decline in firefighters vs. industrial workers [30], one showed a significantly greater rate of decline in general population controls vs. firefighters [32], and four did not report any significant differences in changes in FEV_1_ compared to general population controls [8, 10], emergency medical workers [9] or paper-pulp mill and construction workers [43]. Five studies compared changes in the FVC of firefighters vs non-firefighters with two showing significantly greater FVC declines in firefighters [10, 30], one showing significantly greater FVC declines in non-firefighters [32], and two showing no significant differences [8, 9].

#### Non-routine firefighting

Six studies reported changes in lung function of firefighters exposed to non-routine firefighting [39, 44–48]. Firefighters involved in one study were exposed to smoke during a chemical warehouse fire [39], and experienced declines in FEV_1_ and FVC of − 81.3 and − 41.33 mL/yr., respectively, in the time between measurements after exposure and 18 months later. The remaining five studies reported on the changes in FEV_1_ observed in a cohort of New York firefighters following World Trade Centre site exposure after the terrorist attacks of September 11, 2001 (9/11). The pre-9/11 rate of change in FEV_1_ in firefighters and Emergency Medical Service (EMS) workers was reported as − 31 mL/yr. [44], while each group lost an average of 383 [95% CI, 374–393] mL and 319 [299–340] mL, respectively, in the first year following the disaster. In the 7 years after the initial reduction, the rate of change in FEV_1_ (adjusted for age, height, race and sex) of never-smoking firefighters was − 26 [95% CI, 20–31] mL/yr.: less than that of former or current smokers and significantly different from the − 40 [38–42] mL/yr. observed in never-smoking EMS workers [45]. A similar rate FEV_1_ decline of − 26.4 mL/yr. was observed in a follow-up study of the never-smoking firefighters after 13 years [46]. Compared to continuing smokers, the rate of change in FEV_1_ of former smokers who quit before or after 9/11 was significantly less negative. Two small subgroups of 9/11-responding firefighters were also studied, observing post-9/11 FEV_1_ declines of − 36.7 mL/yr. (adjusted for age, bronchial hyper-reactivity, height, race, steroid use and the initial loss of lung function related to 9/11 exposure) [47] and 37 mL/yr. (adjusted for age, height, interaction of smoking with AAT deficiency, length of FDNY tenure, race, sex, smoking, work assignment on 9/11 and WTC exposure intensity) [48].

In summary, most studies of non-smoking firefighters exposed to routine firefighting showed negative rates of change in FEV_1_ and FVC that were analogous to the rates observed in longitudinal studies of healthy non-smokers in the general population [12–22]. Those that showed greater rates of decline than would normally be expected were either less than [32] or not significantly different to [10] general population controls in direct comparisons, or were particularly limited by a lack of information on smoking status [30, 31, 37]. Firefighters exposed to non-routine events experienced significant reductions in lung function in the initial year after exposure, with long-term rates of change representing normal decline without recovery.

### Influence of firefighting and protective or deleterious factors

#### Influence of firefighting exposure level

In their 1974 report of Boston firefighters, Peters and colleagues showed significant inverse relationships between self-reported fire exposure over a 12-month period and changes in FEV_1_ and FVC [37]. However, no significant relationship was observed in three [35] and six-year [34] follow-up studies on the same population, using self-reported exposure and estimates derived from fire department records. A significantly greater FEV_1_ decline was observed in active vs inactive firefighters in one study [33] but is contrasted by two others which showed trends of higher rates of decline in inactive vs active firefighters [34, 35], while a further study showed no difference [30]. No studies identified a relationship between service time and rate of change in FEV_1_ or FVC [10, 30, 31, 33, 35, 36]. One study reported significantly greater rates of FEV_1_ decline in firefighters who reported previous exposure to ammonia, however past chlorine exposure had no apparent effect [33].

Firefighters responding to the 9/11 disaster experienced dramatic declines in FEV_1_ in the first year following exposure [44–48]. Measured by self-reported arrival time, a significant dose-response relationship was observed between exposure intensity and loss of FEV_1_ [44]. Firefighters that reported the greatest dust exposure (those arriving earliest) also experienced the greatest rate of FEV_1_ decline in the subsequent 7 and 13-year follow-ups [45, 46].

The included studies show a dose-response relationship between changes in lung function and exposure level in non-routine severe firefighting events, but results were inconsistent regarding the presence of such an effect of exposure level in routine firefighting.

#### Influence of respiratory protection

Four studies investigated the effect of respiratory protection on changes in FEV_1_. In one study, firefighters who reported ‘never or rarely’ using their respiratory protection during fire knockdown had higher odds of ‘accelerated’ FEV_1_ decline (greater than 50 mL/yr) compared with those who used it ‘often or frequently’ (Odds Ratio = 2.20, 95% Confidence Interval = 1.02–4.74, *p* = .044) [8]. Another study observed a greater FEV_1_ decline in firefighters who reported ‘never’ vs ‘ever’ using a mask while extinguishing fires (− 68.44 vs − 30.90 mL/yr), but the association was only significant in those with non-repeatable spirometry [33]. There was no significant difference in changes of FEV_1_ based on mask-use during fire overhaul (clean-up). A further study showed no relationship between the rate of change in FEV_1_ and the stated tendency of firefighters to wear protective respiratory apparatus [34] while there was also no identifiable protective effect of using any type of protective mask during the response to the 9/11 disaster [44].

#### Influence of other factors

In the five studies that included covariates in their models to estimate changes in lung function, four included race, sex and smoking status as well as baseline age and height [44–46, 48], while one included only race as well as baseline age and height: due to the absence of females and separate analyses with smokers [9]. Three of these studies included weight at baseline [9, 45, 46] with one also including weight change in a separate model [9]. These five variables as well as a further 20 were included in subsequent modelling to investigate factors that affect the rate of change in lung function (all variables listed in Table 2). Overall, noteworthy predictors included weight gain, which was associated with a significantly greater decline in FEV_1_ in two studies [9, 46] and close to being significant in another [41], while four studies observed significantly increased or decreased rates of FEV_1_ decline based on different variations in gene expressions [40–42, 48]. One study associated the development of bronchial hyper-reactivity with a significant increase in FEV_1_ decline in 9/11-exposed firefighters, while the use of steroids was associated with a less negative rate of change in FEV_1_ [47].

### Calculation/measurement and reporting of the rate of change in FEV_1_ and FVC

Eight studies calculated the rate of change in FEV_1_ and/or FVC as the change in volume divided by the change in time using data from two time-points [8, 10, 33–37, 47]: four of which had measured lung function on more than two occasions [34–36]. Five studies used simple linear regression [32, 40–43], five used linear mixed models [9, 44–46, 48], while a further four did not report on the rate of change, or did not report their method of calculation [30, 31, 38, 39]. There was no apparent indication that any technique was more biased toward positive, negative or null results.

Six studies reported on the proportion of firefighters with a decline in FEV_1_ or FVC that was greater than a particular cut-off: often referred to as an ‘accelerated’ or ‘greater than expected’ decline. The cut-offs (proportion of firefighters above cut-off) were set at declines of > 50 mL/yr. (Fire: 26%, Controls: 39%) [8], > 60 mL/yr. (18.4% [42], 23% [40]), > 64 mL/yr. (19.5%) [46], ,> 75 mL/yr. (50.8%) [38] and > 90 mL/yr. (4.8%) [43], with the latter study also using a relative cut-off of > 2.1% per year (5.6%) [43]. One study also reported on FVC declines of greater than 75 mL/yr. (35.1%) [38].

### Quality assessment/risk of bias

Two articles were rated as high quality/low risk of bias, 12 as moderate quality/moderate risk of bias, and eight as low quality/high risk of bias (Table 3).

**Table 3:**
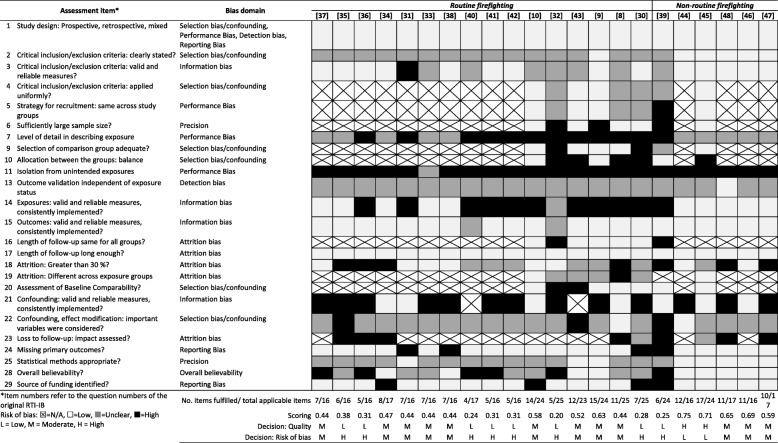
Summary of individual study quality/risk of bias assessment using the RTI-IB. Studies are ordered by population type and year of publication

The most evident biases were performance bias, information bias and attrition bias. Studies generally failed to use valid and reliable means of measuring exposures and did not report them with great detail in respect to the measurement and reporting of confounding variables. Seven studies reported loss to follow-up of greater than 30%, yet none investigated any potential effect of this through sensitivity analyses or other adjustment methods.

Biases that were the most unclear were selection bias/confounding, detection bias, performance bias and precision. The most common issues were around the clarity of inclusion/exclusion criteria, the lack of clarity in reporting blinding of assessors to exposure status as well as the appropriateness of statistical techniques: although this was mainly a reflection of the age of the studies.

## Discussion

To our knowledge, this is the first study to systematically review the literature measuring longitudinal changes in lung function of professional urban firefighters and its associations with occupational exposure. Among firefighters exposed to routine firefighting, the reported rates of change in lung function were variable and ranged from normal rates of decline to what could be considered accelerated: particularly among current smokers. There is a general lack of evidence of a relationship between measures of routine firefighting exposure and long-term changes in lung function: though this may be primarily due to limitations in exposure measurement itself. In contrast, exposure to non-routine disastrous events is more clearly related to reductions in lung function.

The large variability in the reported rates of change in lung function of firefighters exposed to routine firefighting make definitive conclusions difficult. Most observations among never-smokers were consistent with other longitudinal studies of the general population, or were at least no more negative than non-firefighter general population controls. However, the range of findings and low rating in quality assessments among included studies, and the fact that there is no clear upper limit of normal lung function decline, precludes any definitive conclusions about the risks of accelerated longitudinal declines in lung function of professional urban firefighters in relation to routine firefighting.

Among studies of routine firefighters, the study with the highest score (0.63) in the quality rating/lowest risk of bias assessment consisted of 5 years of annual measurements and provides the best evidence of the effect of contemporary firefighting on lung function [9]. Firefighters in this study experienced a longitudinal rate of change in FEV_1_ of − 45 mL/yr. which was equal to that of unexposed controls. While this is greater than the rate of decline reported in most longitudinal studies of healthy adult non-smokers, it is still less than is reported by others such as Tashkin et al. [22] This highlights the difficulties associated with comparing rates of change in lung function between studies of different periods, which utilised different recruitment strategies as well as different equipment and standards of lung function testing. For these reasons, studies that make direct comparisons to a well-matched (yet unexposed) reference group who are sampled in the same way, are particularly valuable, but equally scarce.

One of the few studies employing a control population made a surprising observation of an increase in FEV_1_ and FVC in non-smoking firefighters concurrent to a decline in age-matched, non-smoking general population controls [8]. Although this may be somewhat influenced by the inclusion of younger firefighters whose lungs may still be maturing, the mean changes in FEV_1_ and FVC for firefighters aged 36–45 years were positive. This is contradictory to the notion that lung function declines after peaking during in the third decade of life [15, 21, 49–51]. Caution, though, is needed in interpreting these results, as this study would have benefited from further follow-up in order to reduce any possible effect of statistical regression to the mean.

In attempting to quantify the effect of routine firefighting exposures on changes in lung function, multiple different strategies have been employed, each with limited success. The number of responses to fires has not been meaningfully associated with negative changes in FEV_1_ or FVC, either through estimates based on fire department records (FDR) or recalled by firefighters themselves [33–36]. Musk et al. [35] also reported a poor correlation between the two methods, which may suggest that firefighters cannot accurately recall their exposures over a twelve-month period, that the FDR method of estimation was unreliable, or both. Service/employment time has also been a poor index of exposure [10, 31, 33, 35, 36], and has questionable validity given the way in which firefighters can move between active and inactive roles throughout their careers. This movement of workers also undermines any assumptions that active firefighters have had greater exposures than inactive firefighters, given that firefighters may self-select out of active roles as a direct result of poor health following work-related exposure. Further crude indices of exposure have included self-reported heavy smoke exposure, informally described as “shellackings” [35, 37], “lungers” or “pastings” [34], as well as absence from work following exposure to smoke [31], showing no significant associations with changes in lung function.

Examined separately from studies of firefighters exposed to routine firefighting, studies investigating changes in lung function following severe exposure reveal consistent outcomes of accelerated declines in lung function. Observing firefighters immediately following exposure to a chemical warehouse fire, Unger et al. [39] reported a high average rate of decline in FEV_1_ over the subsequent 18 months. While a lack of pre-exposure data is a limitation of the study, the rate of decline may have even been underestimated, if there were cases of lung function recovery, over the course of follow-up. This may provide an example of how studies involving ‘non-routine events’ could bias the estimate of the rate of change in lung function following the event and supports the separate interpretation of the results in this review. This issue also applies to the studies which followed firefighters after 9/11 [44–48]. In addition, some of these studies included firefighters who retired during follow-up and thereby removing them from firefighting exposures which may further affect estimates of the rate of change in lung function. Notwithstanding these issues, these studies were among the highest quality rated studies with the lowest risk of bias and have benefitted from the presence of several years of pre-exposure data. They provide unequivocal evidence of the dramatic long-term negative effect of this exposure on lung function and highlight the importance of routine lung function surveillance in firefighters.

Among all studies included in the review that made the comparison, most studies observed greater rates of decline in never-smoking firefighters compared to current-smoking firefighters. Although the significance of this difference was not always tested statistically, the excess declines in current smokers were comparable to those observed in general smoking population [52, 53]. Cigarette smoking has the potential to be particularly dangerous to firefighters, given that it has been linked with reductions in immune responses [54], which may leave them more vulnerable to the dangers of fire smoke. Based on the information available in this review, however, it was not possible to speculate any further than this.

Along with smoke exposure, both from fires and cigarettes, one of the most important variables affecting firefighter lung function trajectories is the use of respiratory protection, which has undergone many changes across the time periods of the included studies. The US-based National Fire Protection Association (NFPA) produced its first *Standard for Respiratory Protective Equipment for Firefighters* (*NFPA 19B*) in 1971, with the aim of prohibiting filter-type canister masks for firefighters and permitting only self-contained breathing apparatus (SCBA) [55]. The regularly updated standard has overseen improvements in technology that are likely to have influenced the frequency with which SCBA is utilised by firefighters, which may have implications on respiratory health. In their pioneering studies of the early 1970s Boston, Musk et al.*..* found no relationship between firefighters’ “self-stated tendency” to use respiratory protection and changes in FEV_1_, but provided no further information on the frequency of use [34]. Tepper and colleagues later compared changes in the FEV_1_ of firefighters who reported ‘never’ vs ‘ever’ using a mask while extinguishing fires, showing little association [33]. This method, though, may lack sensitivity due to the use of the broad term ‘ever’, which may have grouped together those who have used it once only, or at every response. Two decades later, Schermer et al [8] showed that firefighters who reported ‘never or rarely’ using respiratory protection during fire suppression were significantly more likely than others to experience greater declines in FEV_1_. They were also less likely to not use respiratory protection during fire overhaul: the period following extinguishment of visible flame, when exposures are still dangerous [56, 57]. These firefighters were also more likely to be older, suggesting a possible cohort effect whereby use of respiratory protection increases with each new generation of firefighters. Among responders to the 9/11 disaster in New York in 2001, there was no identifiable protective effect of using any type of protective mask [44]. However, this is likely due to the fact that most firefighters were entirely unprotected, or wore only a disposable mask in the first 2 days of the event [58].

Studies that received the highest quality assessment/lowest risk of bias scores tended to be among the most recently published studies, and employed more contemporary statistical methods of analysis [9, 44–48]. Among the remaining studies, there was no discernible relationship between publication date and quality. Mixed models approaches offer several advantages over other ‘pre-post’ analyses, with the latter being more susceptible to influence by measurement error. Further, given the natural variability in lung function measurements, studies with more than two measures of lung function over five or more years of follow-up can more precisely and reliably evaluate the rate of change in lung function [18]. Those studies that met this criterion tended to report normal rates of decline in FEV_1_ or FVC. None of the included studies assessed for non-linear changes in lung function.

A limitation of this review was the absence of meta-analytical techniques, which were precluded by the lack of homogeneity across studies published over several decades. The review may also be limited by publication bias, as it did not include evidence that was unpublished or pending publication. Additionally, the minimum follow-up time for studies to be included was 1 year. Given the value of repeated measurements over long periods [18], approximately half of the studies included may be too short to provide truly meaningful insights into the way lung function changes over time. Further, due to the manner in which published data were reported, some data were estimated from graphical figures using computer software, or calculated from the data that were available and this may have reduced the precision of estimates of rate of change. Moreover, the focus of this review was on professional urban firefighters, whose exposures may differ in type, intensity and duration to those of wildland firefighters. Although exposure to wildland firefighting has produced cross-shift [59] and cross-seasonal [60] reductions in lung function, further studies are needed to investigate the long-term effects of such firefighting.

## Conclusions

The data provided by longitudinal studies, which were mostly concerned with FEV_1_, are highly variable and provide an unclear picture of how the rate of change in lung function of firefighters relates to routine exposures and how it compares to the rate of change expected in a non-exposed working-age population. Firefighters who abstain from cigarette smoking and who routinely wear respiratory protection are more likely than otherwise to have a normal rate of decline in lung function. Exposure to catastrophic events, such as 9/11, significantly increases the rate of decline in lung function but there is limited evidence detailing the effect of routine firefighting and future studies will benefit from more robust methods of measuring exposure.

## Abbreviations

9/11: World Trade Center disaster on September 11, 2001
AA: African-American
AAT: Alpha-1 antitrypsin
B: Black
BHR: Bronchial hyper-reactivity
C: Caucasian
CI: 95% Confidence Interval
Cur: Current smokers
CurNS: Current non-smokers
EMS: Emergency Medical Services
ERS: European Respiratory Society
Ev: Ever smokers
F: Female(s)
FDNY: Fire department New York
FDR: Fire department records
FEV_1_: Forced Expiratory Volume in one second
FIRE: Firefighters
For: Former smokers
FVC: Forced vital capacity
GP: General population
HAZMAT: Hazardous materials
HW: Hispanic White
IL-10: Interleukin-10
IL-IRA: Interleukin-1 receptor antagonist
IQR: Inter-quartile range
Knockdown: Fire suppression
LFB: London fire brigade
LLN: Lower limit of normal
NFPA: National Fire Protection Association
NR: Not reported
NWAHS: North-West area health study
OR: Odds ration
Overhaul: Clean-up following fire suppression
PFT: Pulmonary function test
PRISMA: Preferred Reporting Items for Systematic Reviews and Meta-Analyses
PROSPERO: International Prospective Register of Systematic Reviews
RMANOVA: Repeated measures analysis of variance
ROD: Rate of decline
RTI-IB: Research Triangle Institute Item Bank
SCBA: Self-contained breathing apparatus
SD: Standard deviation
SE: Standard error
SNP: Single nucleotide polymorphism
TGβ1: Transforming growth factor β1
TNFα: Tumour necrosis factor-α
USA: The United States of America
W: White
WTC: World Trade Center

## References

[1] Greven FE, Krop EJ, Spithoven JJ, Burger N, Rooyackers JM, Kerstjens HA (2012). Acute respiratory effects in firefighters. Am J Ind Med.

[2] Sheppard D, Distefano S, Morse L, Becker C (1986). Acute effects of routine firefighting on lung function. Am J Ind Med.

[3] Sherman CB, Barnhart S, Miller MF, Segal MR, Aitken M, Schoene R (1989). Firefighting acutely increases airway responsiveness. Am Rev Respir Dis.

[4] Chia KS, Jeyaratnam J, Chan TB, Lim TK (1990). Airway responsiveness of firefighters after smoke exposure. Br J Ind Med.

[5] Banauch GI, Alleyne D, Sanchez R, Olender K, Cohen HW, Weiden M (2003). Persistent hyperreactivity and reactive airway dysfunction in firefighters at the world trade center. Am J Respir Crit Care Med.

[6] Brandt-Rauf PW, Cosman B, Fallon LF Jr, Tarantini T, Idema C. Health hazards of firefighters: acute pulmonary effects after toxic exposures. Br J Ind Med. 1989;46:209–11. 10.1002/ajim.21012. PMC1009756 2930733

[7] Schermer TR, Malbon T, Morgan M, Briggs N, Holton C, Appleton S (2010). Lung function and health status in metropolitan fire-fighters compared to general population controls. Int Arch Occup Environ Health.

[8] Schermer TR, Malbon W, Adams R, Morgan M, Smith M, Crockett AJ. Change in lung function over time in male metropolitan firefighters and general population controls: a 3-year follow-up study. J Occup Health. 2013; 10.1539/joh.12-0189-O 10.1539/joh.12-0189-oa 23796594

[9] Aldrich TK, Ye F, Hall CB, Webber MP, Cohen HW, Dinkels M (2013). Longitudinal pulmonary function in newly hired, non-world trade center-exposed fire department city of New York firefighters: the first 5 years. Chest.

[10] Sparrow D, Bosse R, Rosner B, Weiss ST (1982). The effect of occupational exposure on pulmonary function: a longitudinal evaluation of fire fighters and nonfire fighters. Am Rev Respir Dis.

[11] Quanjer PH, Stanojevic S, Cole TJ, Baur X, Hall GL, Culver BH (2012). Multi-ethnic reference values for spirometry for the 3-95-yr age range: the global lung function 2012 equations. Eur Respir J.

[12] Fletcher C, Peto R. The natural history of chronic airflow obstruction. Br Med J. 1977;1:1645–8. 10.1136/bmj.1.6077.1645. 10.1136/bmj.1.6077.1645 PMC1607732 871704

[13] Tager IB, Segal MR, Speizer FE, Weiss ST (1988). The natural history of forced expiratory volumes. Effect of cigarette smoking and respiratory symptoms. Am Rev Respir Dis.

[14] Lange P, Groth S, Nyboe G, Mortensen J, Appleyard M, Jensen G (1989). Effects of smoking and changes in smoking habits on the decline of FEV_1_. Eur Respir J.

[15] Sherrill D, Lebowitz M, Knudson R, Burrows B (1992). Continuous longitudinal regression equations for pulmonary function measures. Eur Respir J.

[16] Rodriguez BL, Masaki K, Burchfiel C, Curb JD, Fong K-O, Chyou P-H (1994). Pulmonary function decline and 17-year total mortality: the Honolulu heart program. Am J Epidemiol.

[17] James AL, Palmer LJ, Kicic E, Maxwell PS, Lagan SE, Ryan GF (2005). Decline in lung function in the Busselton health study the effects of asthma and cigarette smoking. Am J Respir Crit Care Med.

[18] Wang ML, Avashia BH, Petsonk EL (2006). Interpreting periodic lung function tests in individuals: the relationship between 1-to 5-year and long-term FEV_1_ changes. Chest.

[19] Wang ML, Avashia BH, Petsonk EL (2009). Interpreting longitudinal spirometry: weight gain and other factors affecting the recognition of excessive FEV_1_ decline. Am J Ind Med.

[20] Abramson MJ, Kaushik S, Benke GP, Borg BM, Smith CL, Dharmage SC (2016). Symptoms and lung function decline in a middle-aged cohort of males and females in Australia. Int J Chron Obstruct Pulmon Dis.

[21] Kohansal R, Martinez-Camblor P, Agustí A, Buist AS, Mannino DM, Soriano JB (2009). The natural history of chronic airflow obstruction revisited: an analysis of the Framingham offspring cohort. Am J Respir Crit Care Med.

[22] Tashkin DP, Clark VA, Coulson AH, Simmons M, Bourque LB, Reems C (1984). The UCLA population studies of chronic obstructive respiratory disease: VIII. Effects of smoking cessation on lung function: a prospective study of a free-living population 1–3. Am Rev Respir Dis.

[23] Moher D, Liberati A, Tetzlaff J, Altman DG, Group P. Preferred reporting items for systematic reviews and meta-analyses: the PRISMA statement. PLoS Med. 2009;6:e1000097. 10.1136/bmj.b2535. PMC2707599 19621072

[24] Rohatgi A. WebPlotDigitizer version 4.0. Austin; 2017. Available from: https://zenodo.org/record/1039373#.W4CqYugzaUk.

[25] Viswanathan M, Berkman ND (2012). Development of the RTI item bank on risk of bias and precision of observational studies. J Clin Epidemiol.

[26] Margulis AV, Pladevall M, Riera-Guardia N, Varas-Lorenzo C, Hazell L, Berkman ND (2014). Quality assessment of observational studies in a drug-safety systematic review, comparison of two tools: the Newcastle–Ottawa scale and the RTI item bank. Clin Epidemiol.

[27] Fuentes JP, Armijo Olivo S, Magee DJ, Gross DP (2010). Effectiveness of interferential current therapy in the Management of Musculoskeletal Pain: a systematic review and meta-analysis. Phys Ther.

[28] Fuentes CJ, Armijo-Olivo S, Magee DJ, Gross DP (2011). Effects of exercise therapy on endogenous pain-relieving peptides in musculoskeletal pain: a systematic review. Clin J Pain.

[29] Al-Saleh MA, Armijo-Olivo S, Thie N, Seikaly H, Boulanger P, Wolfaardt J (2012). Morphologic and functional changes in the temporomandibular joint and stomatognathic system after transmandibular surgery in oral and oropharyngeal cancers: systematic review. J Otolaryngol Head Neck Surg.

[30] Choi J-H, Shin J-H, Lee M-Y, Chung I-S (2014). Pulmonary function decline in firefighters and non-firefighters in South Korea. Ann Occup Environ Med.

[31] Douglas DB, Douglas RB, Oakes D, Scott G. Pulmonary function of London firemen. Br J Ind Med. 1985;42:55–8. 10.1136/oem.42.1.55. 10.1136/oem.42.1.55 PMC1007417 3965016

[32] Horsfield K, Guyatt A, Cooper FM, Buckman MP, Cumming G. Lung function in West Sussex firemen: a four year study. Br J Ind Med. 1988;45:116–21. 10.1136/oem.45.2.116. 10.1136/oem.45.2.116 PMC1007955 3342193

[33] Tepper A, Comstock GW, Levine M (1991). A longitudinal study of pulmonary function in fire fighters. Am J Ind Med.

[34] Musk AW, Peters JM, Bernstein L, Rubin C, Monroe CB. Pulmonary function in firefighters: a six-year follow-up in the Boston fire department. Am J Ind Med. 1982;3:3–9. 10.1002/ajim.4700030103. 10.1002/ajim.4700030103 6957148

[35] Musk AW, Peters JM, Wegman DH (1977). Lung function in fire fighters, I: a three year follow-up of active subjects. Am J Public Health.

[36] Musk AW, Petters JM, Wegman DH (1977). Lung function in fire fighters, II: a five year follow-up fo retirees. Am J Public Health.

[37] Peters JM, Theriault GP, Fine LJ, Wegman DH (1974). Chronic effect of fire fighting on pulmonary function. N Engl J Med.

[38] Kales SN, Polyhronopoulos GN, Christiani DC (1997). Medical surveillance of hazardous materials response fire fighters: a two-year prospective study. J Occup Environ Med.

[39] Unger KM, Snow RM, Mestas JM, Miller WC. Smoke inhalation in firemen. Thorax. 1980;35:838–42. 10.1136/thx.35.11.838. 10.1136/thx.35.11.838 PMC471394 7221980

[40] Burgess JL, Fierro MA, Lantz RC, Hysong TA, Fleming JE, Gerkin R (2004). Longitudinal decline in lung function: evaluation of interleukin-10 genetic polymorphisms in firefighters. J Occup Environ Med.

[41] Josyula AB, Kurzius-Spencer M, Littau SR, Yucesoy B, Fleming J, Burgess JL (2007). Cytokine genotype and phenotype effects on lung function decline in firefighters. J Occup Environ Med.

[42] Yucesoy B, Kurzius-Spencer M, Johnson VJ, Fluharty K, Kashon ML, Guerra S (2008). Association of cytokine gene polymorphisms with rate of decline in lung function. J Occup Environ Med.

[43] Hnizdo E (2012). The value of periodic spirometry for early recognition of long-term excessive lung function decline in individuals. J Occup Environ Med.

[44] Banauch GI, Hall C, Weiden M, Cohen HW, Aldrich TK, Christodoulou V (2006). Pulmonary function after exposure to the world trade center collapse in the new York City fire department. Am J Respir Crit Care Med.

[45] Aldrich TK, Gustave J, Hall CB, Cohen HW, Webber MP, Zeig-Owens R (2010). Lung function in rescue workers at the world trade center after 7 years. N Engl J Med.

[46] Aldrich TK, Vossbrinck M, Zeig-Owens R, Hall CB, Schwartz TM, Moir W (2016). Lung function trajectories in WTC-exposed NYC firefighters over 13 years: the roles of smoking and smoking cessation. Chest.

[47] Aldrich TK, Weakley J, Dhar S, Hall CB, Crosse T, Banauch GI (2016). Bronchial reactivity and lung function after world trade center exposure. Chest.

[48] Banauch GI, Brantly M, Izbicki G, Hall C, Shanske A, Chavko R (2010). Accelerated spirometric decline in new York City firefighters with alpha(1)-antitrypsin deficiency. Chest.

[49] Brändli O, Schindler C, Künzli N, Keller R, Perruchoud A (1996). Lung function in healthy never smoking adults: reference values and lower limits of normal of a Swiss population. Thorax.

[50] Van Pelt W, Borsboom G, Rijcken B, Schouten JP, Van Zomeren BC, Quanjer PH (1994). Discrepancies between longitudinal and cross-sectional change in ventilatory function in 12 years of follow-up. Am J Respir Crit Care Med.

[51] Knudson RJ, Lebowitz M, Holberg C, Burrows B (1983). Changes in the normal maximal expiratory flow-volume curve with growth and aging. Am Rev Respir Dis.

[52] Kerstjens H, Rijcken B, Schouten JP, Postma DS. Decline of FEV_1_ by age and smoking status: facts, figures, and fallacies. Thorax. 1997;52:820–7. 10.1136/thx.52.9.820. 10.1136/thx.52.9.820 PMC1758654 9371217

[53] James AL, Palmer LJ, Kicic E, Maxwell PS, Lagan SE, Ryan GF (2005). Decline in lung function in the Busselton health study: the effects of asthma and cigarette smoking. Am J Respir Crit Care Med.

[54] Sopori M (2002). Effects of cigarette smoke on the immune system. Nat Rev Immunol.

[55] NFPA 1981. Standard on open-circuit self-contained breathing apparatus (SCBA) for emergency services. Quincy: National Fire Protection Association; 2007. p. 119.

[56] Bolstad-Johnson DM, Burgess JL, Crutchfield CD, Storment S, Gerkin R, Wilson JR (2000). Characterization of firefighter exposures during fire overhaul. AIHAJ.

[57] Burgess JL, Nanson CJ, Bolstad-Johnson DM, Gerkin R, Hysong TA, Lantz RC (2001). Adverse respiratory effects following overhaul in firefighters. J Occup Environ Med.

[58] Feldman DM, Baron SL, Bernard BP, Lushniak BD, Banauch G, Arcentales N (2004). Symptoms, respirator use, and pulmonary function changes among new York City firefighters responding to the world trade center disaster. Chest.

[59] Gaughan DM, Piacitelli CA, Chen BT, Law BF, Virji MA, Edwards NT (2014). Exposures and cross-shift lung function declines in wildland firefighters. J Occup Environ Hyg.

[60] Liu D, Tager IB, Balmes JR, Harrison RJ (1992). The effect of smoke inhalation on lung function and airway responsiveness in wildland fire fighters. Am Rev Respir Dis.

