# Correction to: The long-term rate of change in lung function in urban professional firefighters: a systematic review

**DOI:** 10.1186/s12890-019-0850-6

**Published:** 2019-05-06

**Authors:** Flynn Slattery, Kylie Johnston, Catherine Paquet, Hunter Bennett, Alan Crockett

**Affiliations:** 1 Alliance for Research in Exercise, Nutrition and Activity, Sansom Institute for Health Research, School of Health Sciences, Universitiy of South Australia, Adelaide, Australia; 2 School of Health Sciences, Sansom Institute for Health Research, University of South Australia, Adelaide, Australia; 3 Centre for Population Health Research, Sansom Institute for Health Research, School of Health Sciences, University of South Australia, Adelaide, Australia

**Correction to: BMC Pulm Med**

**https://doi.org/10.1186/s12890-018-0711-8**

Following publication of the original article [1], the authors flagged an error in Table 2; specifically, within the row beginning ‘Aldrich et al. 2013’ on page 11 of the article PDF.

The error in this row is under the column ‘mL/yr’, and the error reads:

-344.8 [CI, − 347.3 to − 342.3]^i^
-337.6 [CI, − 340.4 to − 334.8]
-336 [CI, − 341 to − 332]

***Please be advised that this data should read:***

-44.8 [CI, − 47.3 to − 42.3]^i^
-37.6 [CI, − 40.4 to − 34.8]
-36 [CI, − 41 to − 32]
